# The Inflammatory Profile of the Tumor Microenvironment, Orchestrated by Cyclooxygenase-2, Promotes Epithelial-Mesenchymal Transition

**DOI:** 10.3389/fonc.2021.686792

**Published:** 2021-06-10

**Authors:** Fernán Gómez-Valenzuela, Enrico Escobar, Ricardo Pérez-Tomás, Viviana P. Montecinos

**Affiliations:** ^1^Department of Hematology‐Oncology, Pontificia Universidad Católica de Chile, Santiago, Chile; ^2^Department of Oral Pathology and Medicine, Faculty of Dentistry, University of Chile, Santiago, Chile; ^3^Department of Pathology and Experimental Therapy - Bellvitge, Faculty of Medicine and Health Sciences, University of Barcelona, Barcelona, Spain

**Keywords:** tumor microenvironment, cyclooxygenase-2, epithelial-mesenchymal transition, cancer, inflammation

## Abstract

The tumor microenvironment (TME) corresponds to a complex and dynamic interconnection between the extracellular matrix and malignant cells and their surrounding stroma composed of immune and mesenchymal cells. The TME has constant cellular communication through cytokines that sustain an inflammatory profile, which favors tumor progression, angiogenesis, cell invasion, and metastasis. Although the epithelial-mesenchymal transition (EMT) represents a relevant metastasis-initiating event that promotes an invasive phenotype in malignant epithelial cells, its relationship with the inflammatory profile of the TME is poorly understood. Previous evidence strongly suggests that cyclooxygenase-2 (COX-2) overexpression, a pro-inflammatory enzyme related to chronic unresolved inflammation, is associated with common EMT-signaling pathways. This review article summarizes how COX-2 overexpression, within the context of the TME, orchestrates the EMT process and promotes initial metastatic-related events.

**Graphical Abstract:**
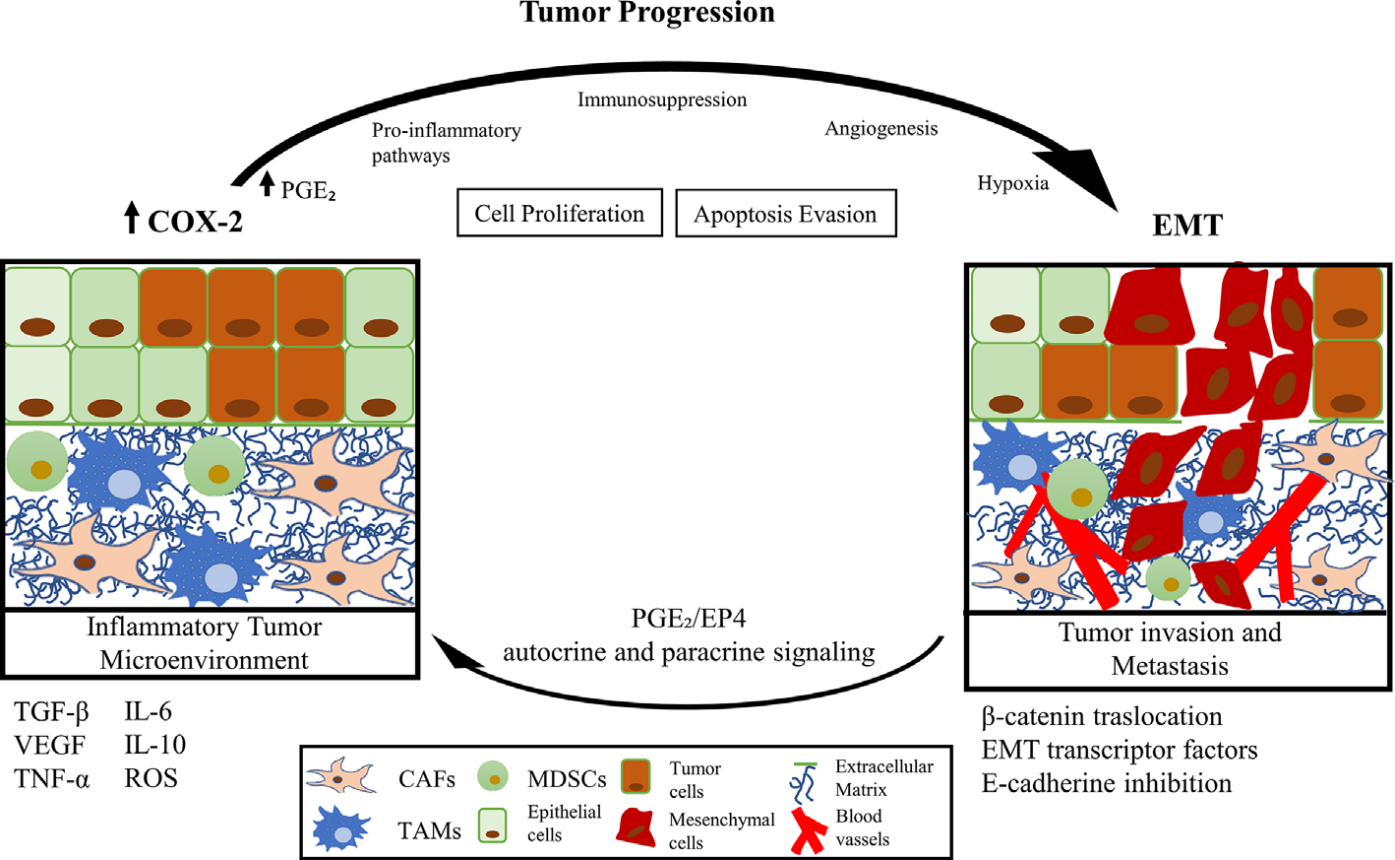


## Introduction

The Epithelial-Mesenchymal Transition (EMT) is a transient and reversible cellular modification program (1), essential for various physiological and pathophysiological processes, like embryonic development, stem cell differentiation, wound repair, and healing (2). EMT is characterized by the transition from an epithelial to mesenchymal cellular phenotype, allowing cell migration and invasion (3). Recent researches showed that EMT corresponds to a partial and transitory cellular event composed of multiple stages (4, 5). The main phenotypic changes of epithelial cells that undergo the EMT phenomenon, are the loss of adherens junctions based on E-cadherin and the disorganization of the basal-apical polarity (6, 7). These phenotypic changes favor the expression of classic mesenchymal cell markers, such as vimentin and N-cadherin, together with the increased expression of various transcription factors related to EMT, which are identified both *in vitro* and *in vivo* (8, 9). Besides the phenotypic changes described, structural and functional extracellular matrix (ECM) changes, mainly caused by cytokines secretion as the transforming growth factor-beta (TGF-β), promote modifications of EMT-related cellular markers expression (1, 9). Therefore, the EMT program strongly depends on the microenvironment properties (2). Nonetheless, the high heterogeneity of the cellular context limits to unravel of the full spectrum of transcription factors that support all the cancer cell modifications on gene expression and their specific biological consequences (1, 4, 10). The main approaches have proposed the existence of master zinc-finger transcription factors of the Snail family, likewise, *Twist* and *Zeb*, as the central directors of EMT, which, along with other transcription factors such as the YAP/TAZ pathway, allow the repression of the epithelial phenotype (1, 2).

**Figure 1:**
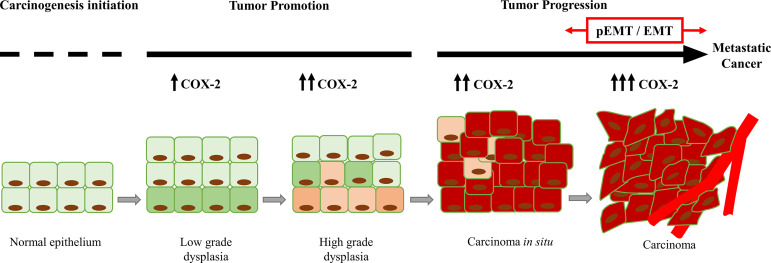
Increase in the COX-2 expression during tumor progression. COX-2 favors tumor progression from the initial stages of tumor promotion (dysplasia). In advanced stages of tumor progression (carcinoma), COX-2 is overexpressed, and it is associated with partial EMT (pEMT) and EMT events.

In the cancer context, tumor cells appropriate the EMT program, which confers metastatic potential (2, 11). Previous works proposed that one of the fundamental characteristics for EMT development could correspond to their inflammatory profile (12–14). Also, the unresolved-chronic inflammation might increase the risk of cancer (10, 12, 15, 16). The presence of TGF-β and other pro-inflammatory cytokines, such as tumor necrosis factor-alpha (TNF-α), interleukin-6 (IL-6), and IL-8, induce the expression of proteins associated with tumor promotion. The overexpression of cyclooxygenase-2 (COX-2), the enzyme responsible for prostanoids synthesis [both thromboxanes and prostaglandins (PGs)], has been indicated as the leading promoter of the inflammatory profile of the TME (17, 18). COX-2 is strongly related to chronic inflammatory events without resolution (19, 20), tumor growth, angiogenesis, cell invasion, metastasis, and chemoresistance, which lead to a low patient survival rate (17, 21, 22). The inducible COX-2 gene is the master switch that activates the inflammatory response (23). COX-2 protein is a critical mediator of inflammation that valuably influences cell proliferation and migration, apoptosis evasion, immunosuppression, tumor angiogenesis, invasion, and metastasis (23–25) **(****Figure 1****)**.

Currently, it is comprehended that tumor cells can transit through a hybrid EMT phenotype, named partial EMT (pEMT) (26). This pEMT phenotype harbors epithelial and mesenchymal characteristics, promoting a vaster invasive capacity of tumor cells and their collective migration through the keeping of cell-cell adherens junctions. Also, pEMT promotes cancer stemness and drug resistance (27, 28). Cancer cells experiencing pEMT are recognized in the early stages of tumor progression (28) and strongly depend on the tumor context (29). Various microenvironment components, like TGF-β, vascular endothelial growth factor (VEGF), and the hypoxia-inducible factor 1 (HIF-1), are responsible for promoting pEMT (30). We suggest consulting the currently published review by Aggarwal et al. (28) to detail analyze the relationship of the TME with the induction and retention of pEMT.

Surprisingly, the mechanisms proposed for the plasticity and maintenance of pEMT are related to the inflammatory cascade resulting from COX-2 activity (30). An approximation of this possible association was described by Lu et al. (31). They suggested that the overexpression of COX-2 in the MCF-10F breast epithelial cell line would induce a partial transformation towards a mesenchymal phenotype. However, the relationship between COX-2 and the maintenance of pEMT state has not been elucidated.

Inflammation is one of the main contributors to the TME (32). Various tumor promoters, oncogenes, and growth factors mediate the COX-2 up-regulation. The COX-2 overexpression has been reported in several human cancers that include breast (23), lung (33), skin (34), esophagus (35), pancreas (36), prostate (37), bladder (38), stomach (39), oral cavity (40, 41), and colorectal adenocarcinoma (19, 42) **(****Table 1****)**. In those studies, COX-2 expression was higher in patients with metastases. For example, Harris et al. indicated that 87% of patients with breast cancer metastases present high expression of COX-2 (23). Furthermore, Huang et al. reported that COX-2 expression is higher in tumor stages T3 and T4 in esophageal cancer, concluding that COX-2 is associated with tumor invasion (35). However, the most specific data that support a cause-and-effect connection between COX-2 and tumorigenesis come from genetic studies in animal models (47). Transgenic mice over-expressing COX-2 in epithelial mammary glands, skin, and stomach develop malignancies and metastatic tumors (48–50). Further, COX-2 overexpression can induce the synthesis of proteases and integrins strongly associated with cancer cell invasion in in different tumor types (51). Thus, COX-2 emerges as a crucial factor for metastatic progression in different types of tumors. Also, COX-2 expression is related to the intracellular pathways activated by phosphatidylinositol 3-kinase (PI3K)/Akt, Wnt/β-catenin, and nuclear factor-kappaB (NF-κB) that are associated with tumor progression. The above suggests COX-2 is a relevant element in promoting EMT by modulating the TGF-β pathway (17, 52) and inducing the decrease or complete loss of E-cadherin expression. Therefore, we propose COX-2 regulates E-cadherin expression indirectly by encouraging NF-κB nuclear translocation, which induces the down-regulation of E-cadherin gene and the expression of EMT transcription factors (5).

**Table 1:** COX‐2 protein overexpression related to tumorigenesis and pro-tumoral activity of epithelial cancers (Refs, references).

System	Histogenesis	Organ	Neoplasm	COX-2 protein overexpression	Biological and (or) Clinical Process	Refs.
**Digestive**	Epithelial	Oral Cavity	Oral Squamous Cell Carcinoma	Promotes the release of PGE2, VEGF, and CD147	Increased Cox-2 expression is associated with the differentiation of human squamous epithelium and is also related to tumor initiation, progression, invasion, and metastasis.	40, 43
**Digestive**	Epithelial	Esophagus	Squamous Cell carcinoma	It was correlated with higher levels of proteins related to cell proliferation, such as Ki-67 and cyclin A. In contrast, p27-staining was negatively correlated with COX-2 Overexpression.	COX-2 overexpression is involved in an early stage of squamous cell carcinogenesis of the esophagus. Besides, COX-2 might regulate cell proliferation and tumorigenesis of esophageal epithelial tumors.	35
**Digestive**	Epithelial	Gastric	Adenocarcinoma	Associated with *Helicobacter pylori* infection and the mutation of tumor suppressor genes and also NF-κB mutation.	COX-2 overexpression promotes the proliferation of gastric cancer cells while inhibiting apoptosis. Also, COX-2 overexpression might promote angiogenesis and lymphatic metastasis, which could be associated with cancer invasion and immunosuppression.	39
**Digestive**	Epithelial	Colorectal	Adenocarcinoma	Induces increase of PGE2 production.	Related to advanced tumor states and correlates with poor clinical outcomes.	19
**Digestive**	Epithelial	Pancreas	Adenocarcinoma	May affect the epidermal growth factor receptor (EGFR) signaling pathway. Prostaglandin synthesis transactivates the induction of EGFR phosphorylation, thereby contributing to pancreatic tumor proliferation. Intrinsic cell role for COX-2 in tumor initiation and progression through activation of the PI3K/AKT pathway.	COX-2 overexpression promotes cell proliferation and is correlated with tumor invasion, angiogenesis, and resistance to apoptosis. COX-2 overexpression correlated with a poor prognosis for patients with pancreatic cancer.	36, 44
**Respiratory**	Epithelial	Lung	Adenocarcinoma	Promotes VEGF, MMP-2, and EGRF expression.	COX-2 overexpression promotes tumor growth, cell infiltration, and metastasis.	33
**Urinary**	Epithelial	Bladder	Urothelial Carcinoma	Suppresses the cytotoxic function of immune cells.	COX-2 overexpression is associated with recurrence and invasion of urothelial cancers, indicating its role as a marker of aggressive behavior.	38, 45
**Endocrine**	Epithelial	Breast	Adenocarcinoma	Induces the transcription of CYP-19 and aromatase-catalyzed estrogen biosynthesis.	COX-2 overexpression is associated with mammary carcinogenesis’s essential features (mutagenesis, mitogenesis, angiogenesis, apoptosis inhibition, metastasis, and immunosuppression).	23
**Endocrine**	Epithelial	Prostate	Adenocarcinoma	It is related to protein kinase C epsilon type (PKCϵ) overexpression. Also, COX-2 inhibits the PTEN pathway, promoting NF-κB activation. Stimulates angiogenesis through the production of prostaglandins and VEGF, which are known as pro-angiogenic factors.	COX-2 expression is higher in metastatic prostate tumors and is linked to poor patient outcomes.	37, 46

Although are known various consequences of COX-2 overexpression in the TME, its relationship with the inhibition of E-cadherin expression and EMT phenomenon is still poorly understood. Throughout this review article, we propose signaling pathways that could dominate the relationship between COX-2 (together with its metabolite PGE2, named COX-2/PGE2 axis) and the promotion of EMT in the TME context.

## The Tumor Microenvironment (TME) and Inflammation

The TME of solid malignant tumors is a complex and dynamic set of cancer cells, the ECM, surrounding blood vessels, tumor-associated stromal cells (comprising many types of immune cells and fibroblasts), and their secreted soluble factors (53, 54). The interactions within the TME are essential for the heterogeneity of cancer, clonal evolution, and the increase of multidrug resistance, which leads to tumor progression and metastasis (55). During tumor progression, cancer cells elude signals associated with recovering the tissue homeostasis (21). The angiogenic nature of TME is a relevant factor in tumor growth. Folkman described that carcinomas *in situ* (less than 0.5 to 1 mm in diameter) would have a nonangiogenic profile (56). However, during tumor growth, a shift towards an inflammatory and hypoxic profile of TME causes a strong dependence on angiogenic activity (21), associated with metastatic growth (56). Thus, the TME represents a complex scaffolding of multiple pro- and anti-inflammatory signals without homeostatic balance.

A wide variety of cells participate in this pathologically inflammatory process, which acquire different behaviors and phenotypes in the TME. Among these cell types, tumor-associated macrophages (TAMs) and carcinoma-associated fibroblasts (CAFs) stand out. Within the TME, the COX-2 overexpression orchestrates this inflammatory profile in various types of solid malignant tumors such as gastric cancer (57), colorectal cancer (58), hepatocellular carcinoma (59), melanoma (60), pancreatic cancer (36), endometrial cancer (61), and squamous cell carcinoma of the neck and head (43). Notably, colorectal adenocarcinoma research has helped understand how the COX-2/PGE2 axis affects carcinogenesis and tumor progression. Sada et al. indicates that COX-2/PGE2 overexpression can be observed early in benign lesions like adenomas (62). Therefore, it seems like the sustained overexpression of the COX-2/PGE2 axis favors adenocarcinoma progression in the context of unresolved chronic inflammation (19).

Current investigations have demonstrated a decrease in tumor progression and normalization of the TME through a selective inhibition of COX-2 using the drug celecoxib (CXB) (51, 63). CXB is an anti-inflammatory drug recognized by the World Health Organization (WHO), which can inhibit COX-2 by blocking its functional activity. Based on the action of CXB, it is possible to propose a dual treatment capable of modulating the inflammatory nature of TME, and concomitant, promote the immune response in anti-tumor therapies. Interestingly, this observation was support by Evrard et al., who indicated that COX-2 co-localizes with PD-1 ligand (PD-L1) in peripheral regions of the tumor and its surrounding inflammatory stroma (64). These observations correlate with previous works that demonstrated a positive relationship between the expression of COX-2 and PD-L1 and a high number of metastatic events to lymph nodes in lung adenocarcinoma (65), which could be crucial to understand immunotherapy resistance (64, 66–68). Furthermore, the development of synergic strategies targeting the immune system and the inflammatory nature of the TME is crucial, considering that EMT dramatically influences the response to anti-tumor therapies (69, 70).

### COX-2 Overexpression in the Non-Tumoral Cells of the TME: TAMs, Cancer Stem Cells (CSCs), Myeloid-Derived Suppressor Cells (MDSCs) and CAFs

**Figure 2:**
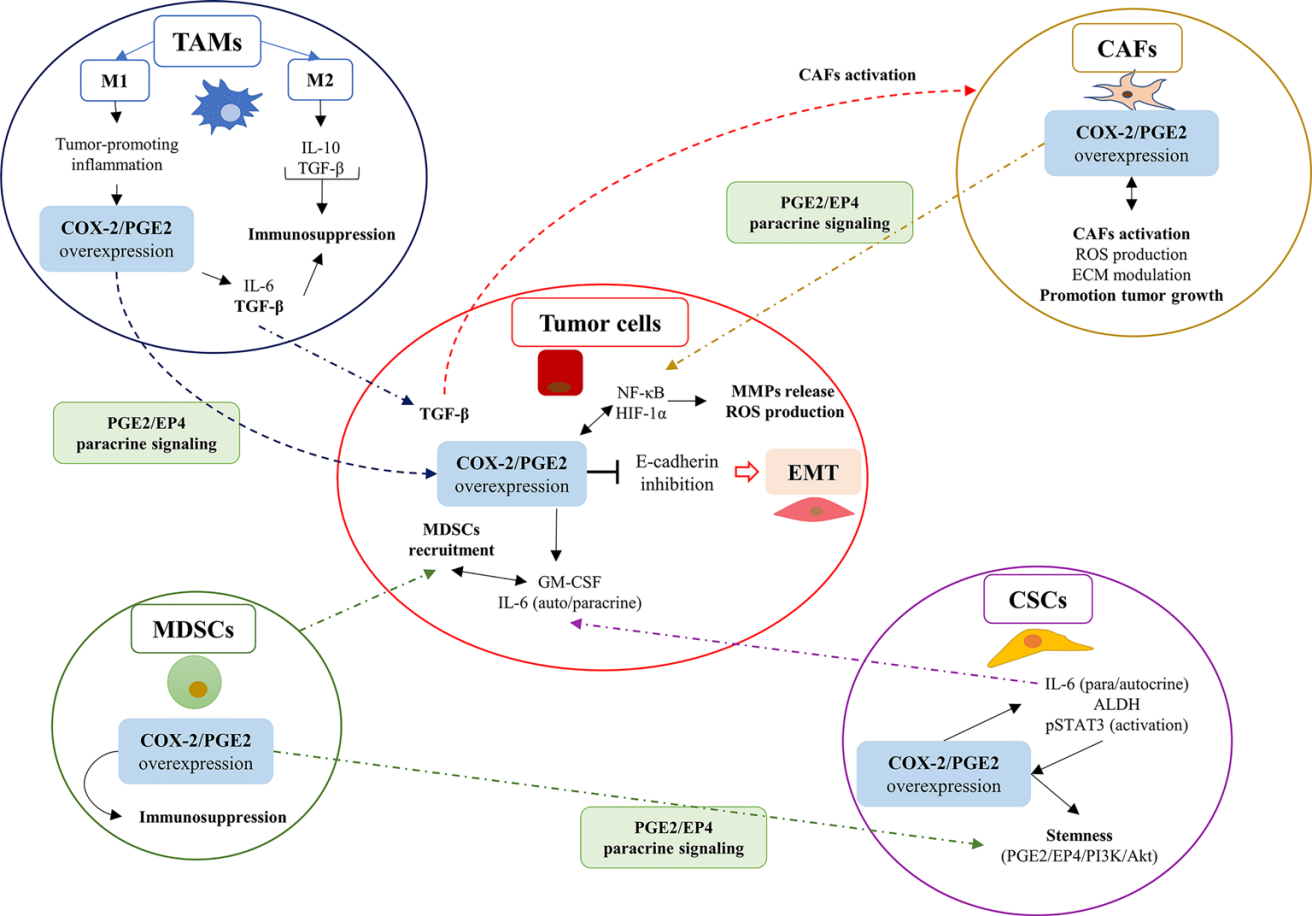
The overexpression of the COX-2/PGE2 axis in cells within the tumor microenvironment (TME) promotes EMT in tumor cells through PGE2/EP4 paracrine signaling. The overexpression of the COX-2/PGE2 axis in cells within the TME promotes EMT in tumor cells through PGE2/EP4 paracrine signaling. The COX-2/PGE2 axis overexpression favors the EMT phenomenon in tumor cells as a consequence of the inhibition of E-cadherin expression. The COX-2/PGE2 overexpression promotes signaling related to ROS and hypoxia, which explains the inflammatory profile of the tumor microenvironment (TME) in solid tumors. Carcinoma-associated fibroblasts (CAFs); cancer stem cells (CSCs); myeloid-derived suppressor cells (MDSCs); and tumor-associated macrophages (TAMs).

Here, we describe the effects of the COX-2/PGE2 axis activity in the TME, and how would induce the EMT program in cancer cells **(****Figure 2****)**.

#### COX-2 Overexpression in TAMs

TAMs, originated by the extravasation of monocytes attracted due to inflammatory cytokines secreted by tumor cells (54, 71), stimulate tumor growth, promote EMT and metastasis (54, 72). COX-2 overexpression occurs in different phenotypes of TAMs, and it is accompanied by the expression of COX-2 in tumor cells (73, 74). Gan et al. showed that TAMs co-cultivated with the breast cancer cell line MDA-MB-231, overexpressed COX-2 and induced cancer cell proliferation through the Akt pathway activation. This effect caused the release of matrix metallopeptidases (MMPs) to the TME and the expression of transcription factors associated with EMT, which down-regulate E-cadherin expression (75). In another study, Han et al. showed that TAMs co-cultured with the osteosarcoma cell lines MG-63 and K-HOS promoted cancer cell migration and invasion *in vitro* and *in vivo* (76). Also, these cell lines showed up-regulation of COX-2 expression, activation of the STAT3 pathway, and increased release of MMP-9 (76). The overexpression of COX-2 in TAMs also up-regulates IL-6, which perpetuates high levels of COX-2 in tumor cells (77). Recent studies proposed that the expression of IL-6 in TAMs could increase the presence CSCs in esophageal cancer, which is associated with an increase of aldehyde dehydrogenases (ALDHs) expression. This mechanism would promote autocrine signaling of IL-6, together with the activation of the STAT3 pathway in CSCs and tumor cells (15, 78).

Nonetheless, one of the biggest challenges for analyzing the nature of TAMs corresponds to the modulation of their polarity (that is, the differentiation between M1 and M2 phenotypes). Despite the association of the M2 phenotype of TAMs with anti-inflammatory signals, subtypes of alternatively activated M2 endotype (AAM) were associate with tumorigenesis and tumor progression (79). One of the M2 subtypes, known as M2a, has been related to the wound healing process and could be associated with EMT through TGF-β signaling (80). This TAMs-M2 subtype also has been associated with an immunosuppressive profile characterized by increased levels and activation of STAT3 (81). Previous works suggested that TAMs polarization signaling is orchestrated by STAT3/6, TGF-β, and NF-κB, along with other pro-inflammatory pathways closely related to the COX-2/PGE2 axis. Therefore, we hypothesized that in cancers with a high inflammatory profile, the coexistence of different polarities of TAMs could generate cellular communications that favor EMT. Interestingly, Zhao et al. informed that the COX-2 overexpression in TAMs causes up-regulation of TGF-β1 on HCT-116 colon cancer cell line through paracrine pathways (82). This observation was associated with increased expression of the response gene to complement 32 (RGC-32) and the promotion of cell proliferation and migration (82). Several studies propose that the phenotype of TAMs represents a crucial element in the reprogramming profile of their immunosuppressive behavior (83). Hence, we emphasized the necessity of new efforts to explain the mechanisms underlying TAMs acquire different phenotypes in cancer. Also, the discovery of new TAMs biomarkers might be helpful for its modulation in immuno-oncology therapies.

#### COX-2 Overexpression in CSCs

COX-2 is overexpressed in CSCs from different types of tumors, which include head and neck squamous cell carcinoma (HNSCC) and non-small cell lung cancer (NSCLC), among others (17). Wu et al. showed that aberrant activation of the COX-2/PGE2 axis promotes stemness through the activation of the Wnt/β-catenin pathway in glioblastoma cells (84). Besides, the presence of CSCs was related to radiotherapy resistance and poor survival outcome (85). Interestingly, Majumder et al. exhibited in a preclinical mouse model of breast cancer cell line MCF-7, CSC markers associated with COX-2 expression and EMT (86). PI3K and Akt pathways were common pathways between COX-2 overexpression and EMT promotion. Moreover, the authors established that the PGE2 receptor EP4 protected these cells from apoptosis (86). This result suggests that COX-2 overexpression would induce genes related to progression of CSCs phenotype during EMT.

Consequently, Tong et al. proposed that this COX-2 overexpression was associated with an increase in the CSCs population in various types of tumors (15). Therefore, it has been proposed that selective inhibition of the EP4 receptor combined with other chemotherapeutic strategies, like endocrine therapies, could be used synergistically against chemo resistant cancers (87). Moreover, Lin et al. showed, in a breast cancer model, that the inhibition of the PGE2/EP4 pathway reduces the chemoresistance and the CSCs population in the TME (88). However, until now, the particular role of EP4 receptor in the EMT program has not been established. Terzuoli et al. demonstrated in metastatic melanoma cell line WM266-4 and the metastatic NSCLC cells HCC4006 a large number of CSCs that regulate the redox state of TME (89). Moreover, these authors reported that ALDH expression was related to a higher expression of the EMT markers, NF-κB factor, and COX-2/PGE2 axis. Also, the authors proposed the ALDH3 blockade could be a helpful therapy for cancers strongly inflamed and associated with immunosuppression and stemness. Moreover, they showed that this strategy could be beneficial when therapies based on anti-PD-1/PD-L1 treatment did not have positive outcomes (78, 89). Lastly, it is relevant to recognize that in cancer cell lines from diverse origins, microRNAs (miRNAs) could promote COX-2 overexpression in CSCs (86, 90–92). This information considers possible alternative pathways beyond those traditionally presented, such as stimulation with lipopolysaccharides (LPS) and TNF-α, that could promote COX-2 overexpression in CSCs. Therefore, new studies examining how the inflammatory nature of TME modulates COX-2 activity in CSCs would complement the currently association between EMT and stemness (16, 93).

#### COX-2 Overexpression in MDSCs

Interestingly, MDSCs, generally described as HLA-DRlow/-CD33+CCD11b+, are not present in normal tissue. Their presence is strongly associated with tumor development and the promotion of immuno-resistance in anti-tumor therapies (94, 95). Similarly, MDSCs are an essential cell type that promotes tumor neovascularization and TME immunosuppression based on CD4+ and CD8+ T cell imbalance (91, 96). Li et al. demonstrated in the nasopharyngeal cancer cell lines TW03 and CNE1, co-cultured with MDSCs, that cell-cell contact stimulated the expression of TGF-β and N2O cytokines in tumor cells (91). Furthermore, the authors illustrated that PGE2 acted on MDSCs EP4 receptors, activating p38-MAPK/extracellular-signal-regulated kinase (ERK) pathways and inducing TGF-β secretion, which causes positive feedback that stimulated EMT. Also, these signals promoted COX-2/PGE2 axis overexpression, as well as β-catenin nuclear translocation (91, 97). On the other hand, Yan et al. confirmed in models of colorectal cancer, that the homeostatic imbalance of TME affected the downstream signal of receptor-interacting protein kinase 3 (RIPK3). RIPK3 was associated with necroptosis promotion, causing the overexpression of the COX-2/PGE2 axis and the induction of the immunosuppressive profile in MDSCs (98). Lastly, Sangaletti et al. suggested that COX-2 overexpression in mammary carcinoma tumor cells, SN25A and SN25ASP activates the IL-6 specifically in rich-collagen stromal areas, inducing the MDSCs recruitment. Further, it was proposed that COX-2 may induce the expression of tumor-associated myelopoiesis factor expression called granulocyte-macrophage colony-stimulating factor (GM-CSF), which allowed cell-cell contact between MDSCs and tumor cells that also favored EMT (99).

#### COX-2 Overexpression in CAFs

Solid tumors which present CAFs in the tumor stroma have a worse prognosis (100). CAFs differ from normal fibroblasts for the expression of smooth muscle α-actin (α-SMA). CAFs have a phenotype characterized by an over-activated proliferative, secretory, and migratory behavior and can perpetuate an enabling framework for EMT development (13, 21, 54, 101) and the progression of solid tumors (102). CAFs impact the structural and secretory profile of the TME, resulting in the loss of tissue homeostasis (13, 21, 102), along with the release of chemo-attractant and immuno-escaping cytokines. Also, CAFs promote the recruitment of myeloid cells, activation and differentiation of MDSCs, and the polarization of TAMs towards the M2 phenotype (13). Despite the notable advance in understanding how CAFs may induce tumor progression, their contribution in the modulation of the COX-2/PGE2 axis is still not fully elucidated. These questions represent a relevant challenge in the understanding of how CAFs modulate the EMT process. Dudás et al. exposed the ability of CAFs to modify the tumor stroma, co-cultivating oral squamous cell carcinoma cell line SCC-25 with fibroblasts derived from the periodontal ligament. This co-culture generated the transformation of fibroblasts to CAFs after stimulation with IL-1β, which also showed a marked nuclear translocation of NF-κB in CAFs. These effects resulted in transcriptomic overexpression of COX-2 in tumor cells (103). Surprisingly, IL-1β stimulation did not generate an increase in the protein expression of inflammatory mediators like IL-6 (103).

Giannoni et al. demonstrated that CAFs represented the primary source of reactive oxygen species (ROS) in TME, which might be related to their marked COX-2 activity. CAFs would activate pro-inflammatory pathways in tumor cells associated with NF-kB/COX-2/PGE2 and HIF-1 activation and, along with it, raising the levels of small GTPase Rac1b. Besides, the authors proposed ROS as the main EMT-inducing factor (102). Alluringly, these events might explain the origin of COX-2 overexpression in TAMs presented by Gan et al., where CAFs might be one of the principal sources of pro-inflammatory agents (75).

Further, Zhu et al. indicated CAFs from samples of nasopharyngeal carcinoma (NPC) present COX-2/PGE2 axis overexpression and correlate positively with TNF-α (104), which together would promote metastasis. However, EMT markers were unfortunately not evaluated in this model (104). Therefore, further studies are needed to propose CAFs, the primary source of COX-2/PGE2 activity of TME, as the main inductor of EMT.

### Repercussions of the COX-2 Overexpression on the EMT Process of Tumor Cells

**Figure 3:**
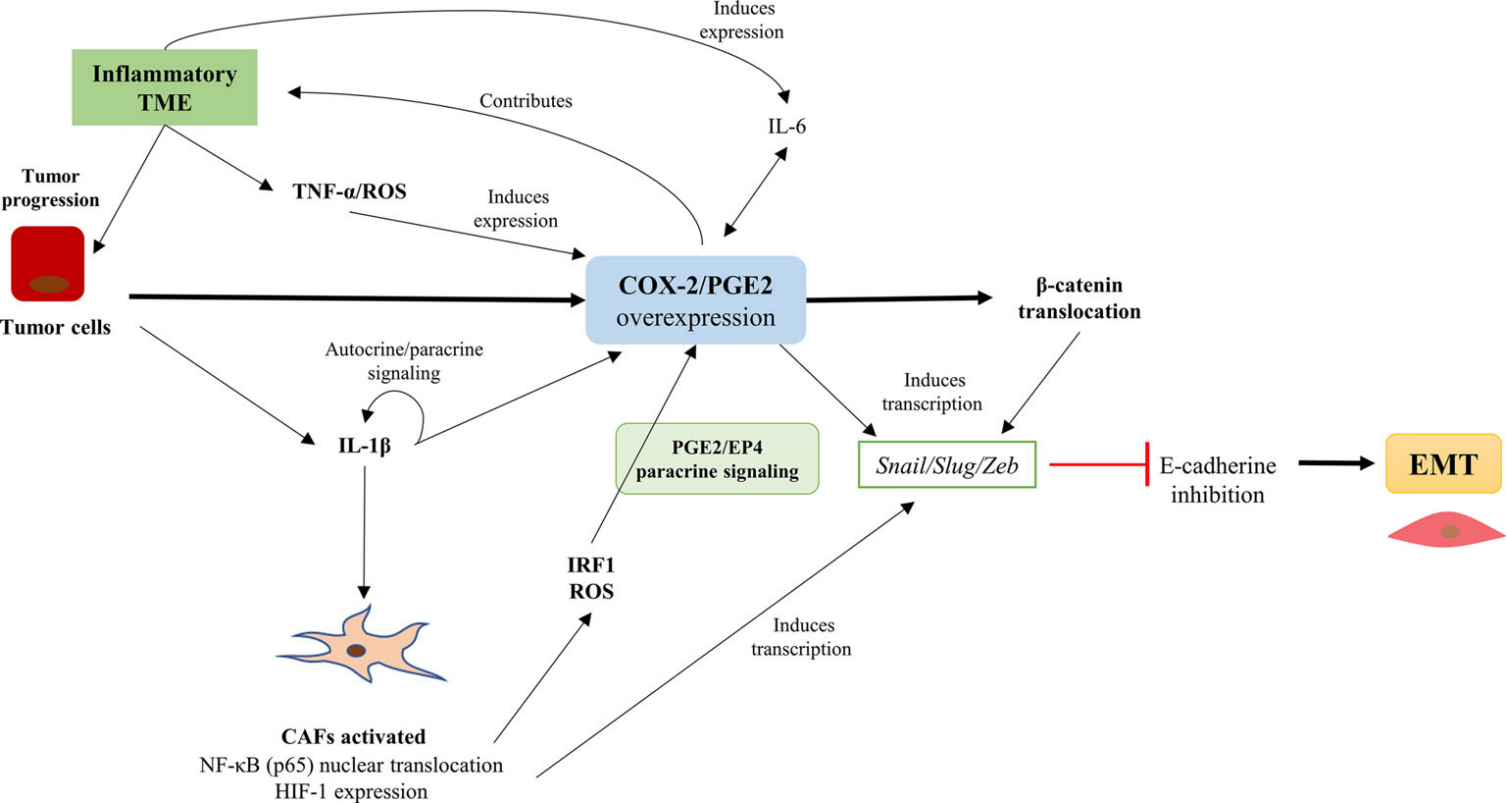
The COX-2/PGE2 axis overexpression is associated with EMT promotion in tumor cells and the presence of cancer-associated fibroblasts (CAFs) in the tumor microenvironment (TME). The COX-2/PGE2 axis overexpression is associated with EMT promotion in cancer cells and the presence of CAFs in the TME. The inflammatory profile of the TME favors the overexpression of the COX-2/PGE2 axis and promotes CAFs activation, which maintain the COX-2/PGE2 axis over-activation. Also, the COX-2/PGE2 axis causing nuclear translocation of β-catenin and the induction of Snail/Slug/Zeb transcription factors expression that repress the E-cadherin and promotes EMT.

COX-2/PGE2 axis overexpression corresponds to a transversal and critical event in the inflammatory nature of TME. Consequently, the stromal cells that orchestrate tumor development promote the over-activation of pro-inflammatory signaling pathways in tumor cells, serving for tumor progression and EMT (**Figure 3**). The evidence suggests a regulation of COX-2 overexpression through a battery of dynamic pro-inflammatory signals emanated from the TME. These pro-inflammatory factors, such as TNF-α and ROS, would stimulate tumor cells directly, affecting COX-2/PGE2 axis overexpression and activation. Furthermore, the same inflammatory context would generate the nuclear translocation of NF-κB and HIF-1, causing an upward rise in these pro-inflammatory factors. The rise of the ROS levels in the TME causes the activation of the interferon regulatory factor 1 (IRF1) in tumor cells and may promote COX-2 activity and PGE2 synthesis (95). In addition, the bidirectional relationship between IL-6 with COX-2/PGE2 overexpression would perpetuate inflammatory events in tumor cells. PGE2 and its receptors play a predominant role in promoting cancer progression. IL-6, also known as interferon-beta2 (IFN-β2), is a pro-inflammatory cytokine that plays a role in inflammation, immune response, hematopoiesis, and cell differentiation. IL-6 production in macrophages is directly stimulated by the COX2/PGE2 axis and TGF-β, while IL-1β and LPS indirectly stimulate IL-6 production *via* NF-κB activation. A positive association exists between endogenous COX-2 metabolites and IL-6 synthesis in both *in vitro* and *in vivo* models of several types of malignant neoplasms (17, 105). IL-6 is the major cytokine in the TME with an extensive range of biological activities. However, the IL-6 expression is deregulated in cancer (106). The IL-6 overexpression is has reported in various cancers, especially from epithelial histogenesis (107) and hematological malignancies (108).

Interestingly, the high IL-6 levels in the TME serve to associate chronic inflammation and cancer progression. IL-6 regulates various cancer hallmarks and multiple signaling pathways, such as JAK/STAT3, Ras/MAPK, and PI3K– PKB/Akt. IL-6 regulates many gene products that cause tumor cell growth, evasion of apoptosis, angiogenesis, metastasis, and immunomodulation of the TME (105). Moreover, IL-6 protects cancer cells from therapy-induced DNA damage, oxidative stress, and apoptosis by facilitating the repair and induction of counter signaling (antioxidant and anti-apoptotic/pro-survival) pathways (105). Thus, IL-6 leads to the dysregulation of a plethora of cellular activities that generally promote tumor progression. Patients with high circulating IL-6 levels are generally associated with a poor prognosis and shorter survival (105), mainly in breast carcinoma (105), stomach adenocarcinoma (109), and ovarian epithelial cancer (110).

## Activation of Pro-Inflammatory Pathways in Tumor Cells in Response to COX-2 Overexpression

**Figure 4:**
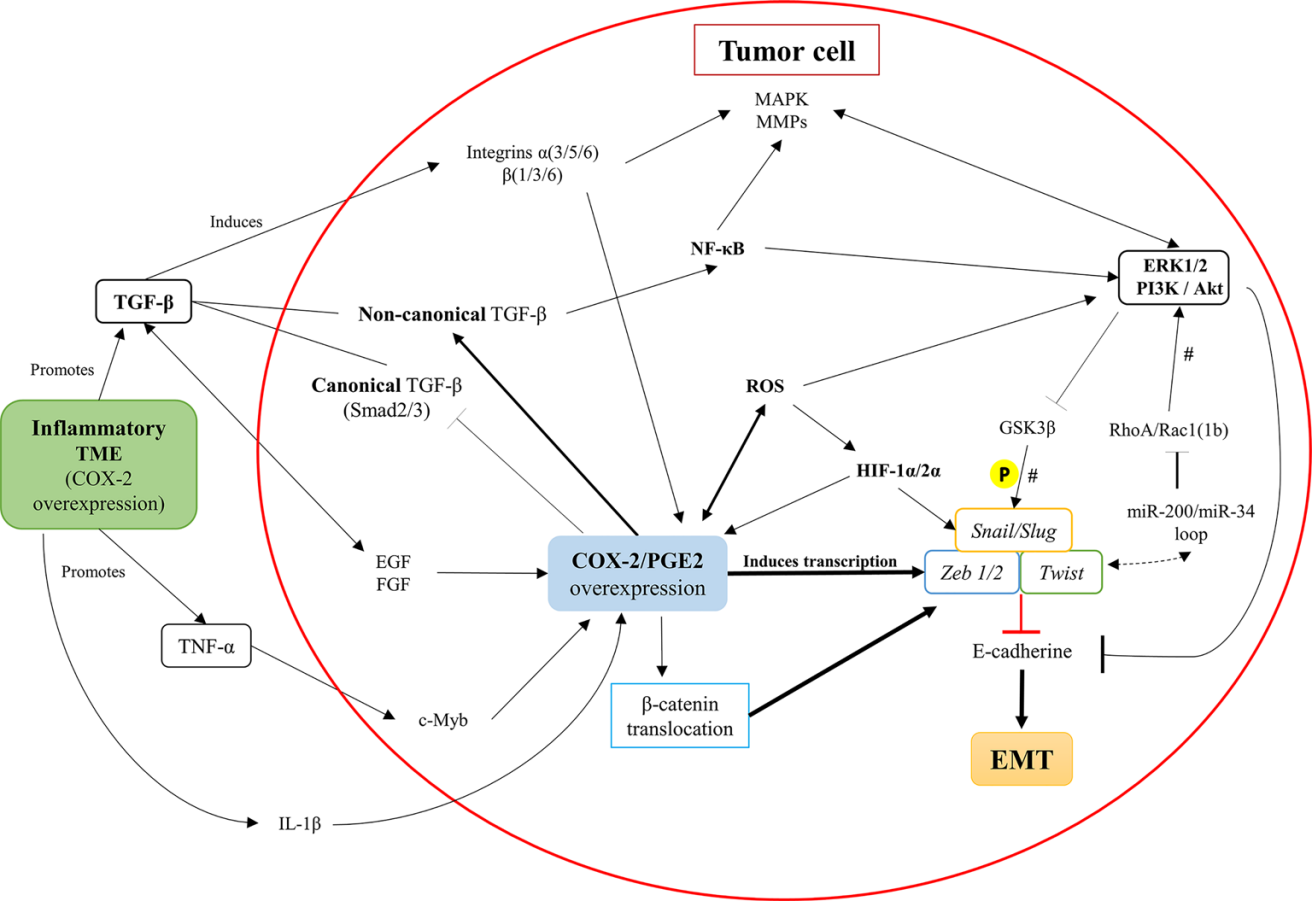
Intracellular pathways in tumor cells that associate COX-2/PGE2 axis overexpression with the promotion of EMT. Inflammatory components of the tumor microenvironment (TME), such as TNF-a and TGF-b, promote the up-regulation of the COX-2/PGE2 axis. Besides, the COX-2/PGE2 axis maintains its autocrine regulation through the activation of the PGE2 EP receptor. The COX-2/PGE2 axis overexpression activates the TGF-β non-canonical pathway (Smad-independent) at the expense of inhibiting the canonical-Smad dependent signaling, along with activation of integrins and secretion of MMPs. Further, the COX-2/PGE2 axis overexpression increases the intracellular ROS levels and activates NF-κB oncogenic functions, which promotes pro-inflammatory signaling pathways and inhibits the GSK3βfactor. The inhibition of GSK3β triggers the activation of master-EMT transcription factors (snail, slug, zeb1/2, and twist), which inhibit E-cadherin expression and finally facilitate the initiation and continuity of the EMT phenomenon.  There is a potential EMT modulation associated with RhoA/Rac1, according to the presence of microRNAs that would maintain a dynamic context-dependent role activating EMT transcription factors. #, their participation is established, but their presence does not determine the process; P, phosphorylation.

As discussed previously, TGF-β is the principal inducer of EMT. However, previous studies have shown that TGF-β can modulate the expression of VEGF, connective tissue growth factor (CTGF), hepatocyte grow factor (HGF), fibroblast growth factor (FGF), and TNF-α. These factors promote the up-regulation of the COX-2/PGE2 axis, resulting in the induction of β-catenin nuclear translocation in cancer cell lines (51, 95, 111–114) and the expression of EMT transcription factors (75, 77, 95, 113). Moreover, COX-2 overexpression increases the invasive capacity of BGC-823 and SGC-7901 gastric cancer cell lines and may be associated with gastric cancer metastasis (114). In addition, COX-2 regulates the mammalian target of Rapamycin (mTOR) factor and could be a helpful prognostic marker in gastric cancer (114)). In summary, **Figure 4** illustrates how the COX-2/PGE2 axis can modulate the classic pro-inflammatory pathways that converge in E-cadherin inhibition and promote the EMT program.

Neil et al. demonstrated in the murine mammary epithelial cell line NMuMG that, after the EMT induction, the COX-2/PGE2 axis overexpression decouples the canonical TGF-β pathway (dependent on Smad2 and Smad3 factors) through glycogen synthase kinase-3 beta (GSK3β). Interestingly, this COX-2/PGE2 axis overexpression promotes its non-canonical (Smad-independent) pathway, determining the aberrant binding of TGF-β with the transcription factor NFκB (52). This activity may equally be related to the stability of β-catenin through GSK3β (15) and transcription factors of EMT (1). Also, it is proposed that the increase of PGE2 expression PI3K/Akt and ERK1/2 pathways, were strongly associated with oncogenic signaling and EMT promotion (15, 52).

These observations turn out to agree with the results of Chen et al. and Liu et al. (115, 116). They demonstrated NFκB translocation in the gastric cancer cell line SGC-7901 due to COX-2 overexpression, which down.regulates E-cadherin expression *via Snail*. Along with this, they observed that the silencing of COX-2 increased the mRNA and protein expression of E-cadherin and *Snail* (115, 116). Furthermore, their results were emphatic for proposing that the functional expression of COX-2 was necessary to regulate NFκB and *Snail* signals in gastric cancer (116). These results support the idea proposed by García de Herreros et al., who identified NF-κB translocation as a relevant inductor of EMT transcription factors expression and E-cadherin repression (5). However, these authors did not provide a discussion about the origins of this phenomenon beyond the presence of TGF-β (5). Dinicola et al. demonstrated in Caco-2 and HCT-8 colon cancer cells that COX-2/PGE2 axis overexpression induces the activation of PI3K/Akt pathway, and the concomitant NF-κB nuclear translocation, promoting invasiveness (113). In addition, they evinced that after selective COX-2 inhibition, E-cadherin levels normalize consistently with previous studies (113). Furthermore, there is a close relationship between NF-κB nuclear translocation and the redox metabolism of cells within the TME, emphasizing its association in tumor progression, EMT, and metastasis (88, 95).

As previously mentioned, another relevant event associated with COX-2 overexpression corresponds to the presence of a high ROS index within the TME. Concordantly, Giannoni et al. showed high ROS levels induce NF-kB nuclear translocation that activates the HIF-1 and Rac1b factors to promote a TME favorable for tumor progression (102). Current studies indicate that HIF-1 is a relevant factor in the activation of EMT and the promotion of the hypoxic and inflammatory profile of the TME (117). One of the central rationales indicates that this association could promote the inflammatory cytokine-induced c-Myb association towards areas of the promoter-type I collagen gene (COL1A2). This event could generate an increase in the stromal component of the TME and promote hypoxic areas with high expression of HIF1-α, proposing the independent analysis of the COX-2 and c-Myb expression (110).

Various studies have described the role of microenvironmental factors such as hypoxia in COX-2 regulation (118). HIF-1alpha (HIF-1α) is a dimeric protein complex that plays an integral role in low oxygen concentrations (119). Likewise, HIF-1α is a significant regulator of oxygen homeostasis within cells (119), activating multiple genes involved in vasodilatation, angiogenesis, neovascularization, cell survival, invasion, and tumor metastasis (120). HIF-1α protein is overexpressed in multiple types of human cancer, including lung, prostate, breast, pancreas, colon carcinoma, and regional and distant metastases (120, 121). Also, overexpression of HIF-1α can occur very early in carcinogenesis (120, 121). COX-2 up-regulation may facilitate adaptation to cellular stress imposed by hypoxia (118).

During hypoxia, COX-2 upregulation results in higher levels of PGE2 (118). PGE2 can enhance HIF-1 transcriptional activity and VEGF induction under hypoxic conditions (119). For example, in hypoxic colorectal tumor cells, high levels of PGE2 enhance VEGF expression and HIF-1 transcriptional activity by activating the mitogen-activated protein kinase (MAPK) pathway, showing a potential positive feedback loop that contributes to COX-2 up-regulation during hypoxia (118). Further, HIF-1α activates the transcription of genes encoding transferrin, VEGF, endothelin-1, and inducible nitric oxide synthase (NOS2), which are implicated in vasodilation, neovascularization, and tumor metastasis (121).

Interestingly, Basudhar et al. showed a NOS2/COX2 crosstalk during tumor promotion and progression (122). Thereby, the TME promotes various inflammatory pathways that induce NOS2 activation and the increase of nitric oxide (NO) levels. This increase of NO lead to COX-2 activity and the synthesis of PGE2, which also induces NOS2 activity and promotes angiogenesis, immunosuppression, and escape from immunosurveillance (122). Therefore, the products of both enzymes mutually regulate each other in such a way that low and moderate amounts of NO are associated with tumor invasion and EMT (123).

Besides, hypoxia promotes the inflammatory cytokine-induced c-Myb expression towards areas of the promoter-type I collagen gene (*COL1A2*). This event could generate an excessive increase of the stromal component in the TME and promote hypoxic areas with high expression of HIF1-α (124). Therefore, we suggest to analyzing in detail the association between c-Myb expression and COX-2.

Lastly, Echizen et al., proposed that the COX-2/PGE2 axis overexpression in macrophages and bone marrow-derived cells (BMDCs) up-regulates the transcription factor noxo1 in tumor cells from colon adenocarcinoma. This signaling was due to the TNF-a pathway and may generate an increase in ROS production and stemness in gastrointestinal cancer cell lines (125). Notably, Huang et al. demonstrated in the oxaliplatin-resistant HT29 colorectal cancer cell line that the alteration of PGE2/EP4 signaling reduces intracellular ROS level and inhibits stemness. Therefore, the overexpression of the COX-2/PGE2 axis could promote chemoresistance and the cellular capacity to counteract the potential damages by and elevated level of intracellular ROS (126). Nevertheless, more studies are needed to evaluate this adaptative mechanism against redox state with the EMT program in tumor cells.

## Epigenetic Modulation of COX-2/PGE2 Axis and the EMT Transcription Factors

The COX-2/PGE2 axis represents a target for epigenetic modifications that allow its over-activation and perpetuates the inflammatory nature of TME (104, 127, 128). Previous studies suggested various miRNAs that can influence the generation of a dynamic and favorable context for EMT development (128). Surprisingly, several studies highlighted a considerable diversity of influences of these miRNAs on the COX-2/PGE2 axis, which is heavily cancer- and cell context-dependent. It is possible to group these results according to how they finally affect the EMT process. For example, there is evidence of EMT inhibition in bladder cancer (129), oral cancer (130), esophageal squamous cell carcinoma (131), lung cancer (132) and NSCLC (133), cervical cancer (134), melanoma (135), and pancreatic cancer (136), among others. Majumder et al., using the breast cancer cell line MCF-7 observed stimulation of EMT by the miR-526b (86). Therefore, it is necessary to recognize its implications according to each case (89). Consistently, robust evidence indicates epigenetic regulation of the COX-2/PGE2 axis, based on miRNAs presence. Moreover, it is crucial to consider that miRNAs also regulate EMT by inflammatory pathways, directly affecting the canonical and non-canonical signaling of TGF-βand indirectly modulating EMT (127).

According to Mishan et al., there are various routes by which miRNAs could promote the EMT program. Concordantly, MiR-145, miR-335, miR-222, miR-150, and miR-34a have been evidence as EMT promoters. Along with this, miR-200 and miR-34 would be part of an EMT regulatory network (128). These miRNAs are strongly associated with the maintenance of the epithelial phenotype. However, as a result of signals such as NF-κB, TGF-β, and HIF-1 in the cancer context, they also promote the activation of the snail and Zeb transcription factors families. This activation allows the up-regulation of the Rac1/RhoA and PI3K pathways (137), which would indicate the promotion of the mesenchymal phenotype necessary for cell migration. Therefore, it is necessary to evaluate epigenetic elements as possible therapeutic targets, notably considering modulate the COX-2/PGE2 axis and could perpetuate the EMT or pEMT program.

## Discussion

Throughout this work, we have highlighted that Epithelial-Mesenchymal Transition is a complex process regulated by microenvironmental demands rather than the internal signaling of each tumor cell. The evidence collected in this work recognizes the COX-2/PGE2 axis as the main driver of EMT and pEMT. The COX-2/PGE2 axis overexpression has an essential role in activating signaling pathways associated with most processes that induce tumor growth and metastasis. Moreover, we consider the COX-2/PGE2 axis not only as a great promoter of tumor progression but metastasis-initiating processes, like EMT. Consequently, it is necessary to carry out clinical studies that consider the potential inhibition of the COX-2/PGE2 axis and EMT markers as metastasis inhibitory treatments in oncology.

Current therapeutic strategies directed to repress the COX-2 overexpression are focused on the inhibition of metabolites resulting from the activity of the COX-2/PGE2 axis. Thus, the specific blockade of the EP4 receptor, has been proposed, showing better results when is with chemotherapeutic regimens or selective COX-2 blocking through CXB (88, 127). Despite this, the selective COX-2 inhibitors have been extensively investigated in diverse types of cancer, generating promising effects as adjuvant therapy. To be specific, it was recognized that CXB reduces the risk of the malignant transformation of colon adenomas towards adenocarcinomas in familial adenomatous polyposis (138). Interestingly, Egashira et al. demonstrated in the HCT-116 colon cancer cell line that CXB inhibits the Wnt/β-catenin signaling pathway, further reducing recurrence after colectomy (139). However, COX-2 inhibitors have been questioned for their potential risk of cardiovascular adverse events. Auspiciously, recent studies showed that CXB has better results according to their gastrointestinal and renal security than other NSAIDs such as ibuprofen and naproxen (140, 141). Also, CXB is associated with fewer cardiovascular adverse events than other COX-2 selective inhibitors, such as rofecoxib (142).

Hashemi Goradel et al. illustrated factors that can modulate the COX-2 targeted therapy, such as type of cancer, type of COX-2 inhibitor, the dose of COX-2 inhibitor, among others (17). Surprisingly, these factors might be associated with the heterogeneous results shown by clinical trials that used CXB as an anti-tumor adjuvant. For example, Hamy et al., based on the exploratory analysis of the REMAGUS02 Trial, indicated that CXB improves the overall survival (OS) and event-free progression (EFP) in breast cancer patients only with high COX-2 expression. Additionally, they indicated the importance of estrogen-receptor status previously the CXB administration (142). Guo et al. demonstrated that the platinum and fluorouracil therapy combined with CXB improved the OS and EFP in patients with advanced and recurrent gastric cancer with COX-2 overexpression (143). However, Kelly et al. evidenced in BOXIT randomized phase III Clinical Trial that CXB does not improve recurrence-free rates of the standard treatment against transitional bladder cell carcinoma (144). Similar results were presented by Bi et al. in Phase II randomized Clinical Trial among patients with NSCLC, without evidence differences between concurrent chemoradiation with or without CXB administration (145). The heterogeneous results of CXB effects in these Clinical Trials could be related to distinct signaling pathways altered in each type of cancer. For example, Zhang et al. showed that the crucial signaling altered in NSCLC was EGF receptor (EGFR). Also, they demonstrated that the use of CXB improves the overall response rate (ORR) (146). Therefore, more studies are required to describe the best therapeutic regimen according to the potential synergism between chemotherapeutic or immunotherapeutic strategies with CXB as an adjuvant.

Other therapeutic strategies with promising prospects are using the association of COX-2 inhibitors and immune checkpoint blockade as a potential choice (147). Several clinical trials of COX-2 inhibitor combination therapy with immune checkpoint inhibitors in cancers have been described. Specifically, the combinations of COX-2 inhibitor inhibitors and anti-programmed cell death protein-1 (anti-PD-1) immunotherapy can promote tumor regression. Wang et al. demonstrated that using COX-2 inhibitor with anti-PD-1 antibody, the objective response rate at six months significantly improved in patients with metastatic melanoma and NSCLC, compared with the anti-PD-1 strategy (148). Furthermore, inhibitor use appears to reverse the unfavorable prognostic effect of a high neutrophil-lymphocyte ratio by prolonging time-to-progression in patients with melanoma (148). Concordantly, Shimizu et al. demonstrated in resected tissue specimens of lung adenocarcinoma that PD-L1 correlated with COX-2 expression, and most cancer cells that express PD-L1 also co-expressed COX-2. However, COX-2 inhibition did not impact PD-L1 expression in NSCLC cell lines as assessed *in vitro*. These experimental results can be interpreted as suggesting that COX-2 inhibition does not affect PD-L1 expression (149). Therefore, COX-2 inhibition possesses little influence of the efficacy of immune checkpoint inhibitors in lung cancer treatment. Despite this, other studies have shown encouraging results for this combined therapeutic strategy in breast cancer and melanoma (68, 150). News perspectives for the combined use (COX-2 inhibitor and immune checkpoint inhibitors) as a practical therapeutic strategy should be sustained with more evidence in the future.

In conclusion, we propose that COX-2/PGE2 axis overexpression should be recognized as an initial promoter of metastasis. The COX-2/PGE2 axis corresponds to the main promotor of the inflammatory profile of the tumor microenvironment and tumor progression. Hence, its inhibition associated with other therapies may provide beneficial results, especially for those tumors that present overexpression of COX-2. Its potential inhibition could mean the reversal of the EMT or pEMT phenomenon and the impediment of metastases.

## Author Contributions

All authors contributed equally to the conception of the work. FG-V drafted and organized the manuscript. All authors contributed to the article and approved the submitted version.

## Funding

This work was partially supported by Project FONDECYT 1150397 (VM).

## Conflict of Interest

The authors declare that the research was conducted in the absence of any commercial or financial relationships that could be construed as a potential conflict of interest.

## Glossary

**Table:**

α-SMA	alpha-actin of smooth muscle
AAM	alternatively activated M2 endotype
ALDHs	aldehyde dehydrogenases
BMDCs	bone marrow-derived cells
CAFs	carcinoma-associated fibroblasts
COL1A2	type I collagen gene
COX-2	cyclooxygenase-2
CSCs	cancer stem cells
CTGF	connective tissue growth factor
CXB	Celecoxib
ECM	extracellular matrix
EFP	event-free progression
EGF	epidermal growth factor
EGFR	epidermal growth factor receptor
EMT	epithelial-mesenchymal transition
ERK	extracellular-signal-regulated kinase
FGF	fibroblast grow factor
GM-CSF	granulocyte-macrophage colony-stimulating factor
GSK3β	glycogen synthase kinase 3 beta
HGF	hepatocyte grow factor
HIF-1	hypoxia-inducible factor-1
HNSCC	head and neck squamous cell carcinoma
IFN-β2	interferon-beta2
IL	interleukin
IRF1	interferon regulatory factor 1
LPS	lipopolysaccharides
MAPK	mitogen-activated protein kinase
MDSCs	myeloid-derived suppressor cells
MMPs	matrix metallopeptidases
miRNA	microRNA
mTOR	mammalian target of Rapamycin
NF-κB	nuclear factor-kappaB
NO	nitric oxide
NPC	nasopharyngeal carcinoma
NSAID	non-steroidal anti-inflammatory drug
NSCLC	non-small cell lung cancer
ORR	overall response rate
PD-1	programmed death protein-1
PD-L1	programmed death protein-1 ligand
PGs	prostaglandins
pEMT	partial epithelial-mesenchymal transition
PKCϵ	protein kinase C epsilon type
PI3K	phosphatidylinositol 3-kinase
RGC-32	complement response gene 32
RIPK3	receptor-interacting protein kinase 3
ROS	reactive oxygen species
STAT3	signal transducer and activator of transcription 3
TAMs	tumor-associated macrophages
TGF-β	transforming growth factor-beta
TME	tumor microenvironment
TNF-α	tumor necrosis factor-alpha
VEGF	vascular endothelial growth factor
WHO	World Health Organization.
